# Systematic human rights violations, traumatic events, daily stressors and mental health of Rohingya refugees in Bangladesh

**DOI:** 10.1186/s13031-020-00306-9

**Published:** 2020-08-20

**Authors:** Andrew Riley, Yasmin Akther, Mohammed Noor, Rahmat Ali, Courtney Welton-Mitchell

**Affiliations:** Colorado School of Public Health and Natural Hazards Center, University of Colorado, Boulder, USA

**Keywords:** Rohingya, Mental health, Human rights violations, Trauma, Daily stressors, Refugees, Bangladesh, Southeast Asia

## Abstract

**Background:**

Almost 900,000 Rohingya refugees currently reside in refugee camps in Southeastern Bangladesh. Prior to fleeing Myanmar, Rohingya experienced years of systematic human rights violations, in addition to other historical and more recent traumatic events such as the burning of their villages and murder of family members, friends and neighbors. Currently, many Rohingya struggle to meet basic needs in refugee camps in Bangladesh and face mental health-related concerns that appear linked to such challenges. The purpose of this study is to describe systematic human rights violations, traumatic events, daily stressors, and mental health symptoms and to examine relationships between these factors.

**Methods:**

Cross-sectional data was collected from a representative sample of 495 Rohingya refugee adults residing in camps in Bangladesh in July and August of 2018.

**Results:**

Respondents reported high levels of systematic human rights violations in Myanmar, including restrictions related to expressing thoughts, meeting in groups, travel, religious practices, education, marriage, childbirth, healthcare, and more. Events experienced in Myanmar included exposure to gunfire (99%), destruction of their homes (93%), witnessing dead bodies (92%), torture (56%), forced labor (49%), sexual assault (33%), and other events. More than half (61%) of participants endorsed mental health symptom levels typically indicative of PTSD, and more than two thirds (84%) endorsed levels indicative of emotional distress (symptoms of anxiety and depression). Historic systematic human rights violations, traumatic events, and daily stressors were associated with symptoms of posttraumatic stress, as well as depression and anxiety. Respondents reported numerous stressors associated with current life in the camps in Bangladesh as well as previous stressors, such as harassment, encountered in Myanmar.

**Conclusions:**

Findings underscore the impact of systematic human rights violations, targeted violence, and daily stressors on the mental health of Rohingya in Bangladesh. Those working with Rohingya should consider the role of such factors in contributing to poor mental health. This research has the potential to inform interventions targeting such elements. Future research should examine the relationships between mental health and human rights violations over time.

## Background

The Rohingya are native to Myanmar’s Rakhine State. Throughout the last several decades, life for the Rohingya in Myanmar has been increasingly characterized by systematic deprivation and human rights violations, with official state policies in place to restrict Rohingya in their ability to marry, travel, have children, access medical care, attend schools, and more [1, 2]. For example, in 2005 a policy was introduced limiting Rohingya families to two children [3]. During a severe wave of violence in 2017, more than 200 Rohingya villages were destroyed, resulting in more than 700,000 Rohingya fleeing into neighboring Bangladesh [4–6]. There, they joined thousands of other Rohingya refugees already living in camps in southeastern Bangladesh as a result of past waves of violence and oppression. The most recent refugees are concentrated in the Balukhali/Kutupalong mega camp, which is now the largest refugee camp in the world [7].

There is a dearth of research examining the mental health implications for Rohingya of exposure to systematic human rights violations over the past several years. In addition, there is very little literature on the mental health impacts of exposure to other potentially traumatic events, and/or daily stressors encountered in the refugee camps for the Rohingya. Until recently, only a few studies focused on mental health of Rohingya [8]. One study in 2013, involving registered Rohingya in camps in Bangladesh, found high levels of daily stressors associated with life in the camps as well as exposure to numerous historical traumatic stressors. Daily chronic stressors associated with life in exile contributed to poor mental health outcomes, specifically symptoms of PTSD and depression [9].. More recently, an assessment conducted by the United Nations High Commissioner for Refugees (UNHCR) similarly identified high rates of acute stress reactions, grief reactions, and post-traumatic stress symptoms in newly arrived Rohingya refugees [8]. In line with this, a qualitative study conducted by International Organization for Migration (IOM) in 2018 indicated that a large portion of the population regularly felt “sad and tense” [10]. In a 2019 journal special issue focused on mental health and psychosocial wellbeing of Rohingya, additional articles highlighted high rates of distress among Rohingya adolescents in Bangladesh [11, 12], and emotional distress among Rohingya in Malaysia [13], in addition to family conflict that appeared to be exacerbated by daily stressors [14]. Despite these recent studies, gaps remain in our understanding of what the Rohingya have endured and how this has impacted their wellbeing.

Historically, symptoms associated with PTSD have been perceived as resulting primarily from an individual’s exposure to specific types of traumatic stressors or life events. However, more recently, a body of research has begun to emerge linking ongoing daily stressors, such as those associated with life in refugee camps, to PTSD symptoms and other forms of distress [9, 15, 16]. Despite this, systematic human rights violations have not been thoroughly examined as potential contributors to poor mental health outcomes for refugees and other displaced populations. This may be in part because chronic systematic human rights violations (e.g., policies restricting the ability to marry, have children, or travel) are not typically included in existing inventories designed to measure exposure to traumatic stress. The purpose of this study is to describe systematic human rights violations, traumatic events, daily stressors, and mental health symptoms, and to examine associations between these factors. This study represents a novel contribution to the literature on refugee mental health, in part because systematic human rights violations have not been thoroughly examined in studies focused on the mental health of refugees and other displaced populations.

## Methods

### Participants, sampling, procedures

Interviews were conducted in person with a randomly selected sample of Rohingya refugee adults (*N* = 495), representative of the adult Rohingya refugee population in the camps in Bangladesh. Interviews were conducted in July and August of 2018. Because there was no comprehensive database of the Rohingya refugee population available, this study used multi-stage cluster sampling to select a representative sample. Thirty-three blocks were randomly selected from a database of all refugee camp blocks, using probability proportionate to size (PPS) to select the blocks for inclusion.

Project coordinators travelled to each selected block to meet with the local block leader (majhi), explain the purpose of the study, and request access to the block-level household lists for the random selection of households within the block. Fifteen households from each block were randomly selected. Of these, eight were randomly assigned as households to survey women, and seven were assigned as households to interview men, matching the gender ratio in the camps. If multiple adults of the selected gender were present in the home, a participant was randomly selected from those eligible using a random number generator application on field researcher smartphones. If no participants of the selected gender were available in the household, field researchers would continue to the adjacent dwelling until they found a respondent of the selected gender.

Surveys took place in refugee homes and took approximately an hour to complete. All surveys were conducted in Rohingya language, and visual aid scales were used to assist in the comprehension of response options. Following completion of surveys in each block, one respondent was randomly selected to participate in a short follow-up survey to ask about their experience participating in the research. This was done to confirm that the data collection procedure was operating according to plan, as well as to ensure that participants felt respected, understood the survey, and did not have any complaints. Most of the field researchers and project coordinators (8 of 10) had previous mental health experience and some had previous research experience. Comprehensive training for the team was provided prior to data collection.

### Survey development and piloting

Data was collected on mobile devices using the Qualtrics offline survey app, with questions administered in-person at the household level by Rohingya members of the research team. Survey data included demographics, systematic human rights violations, exposure to traumatic events, daily stressors, mental health symptoms and functional impairment. Standardized measures were adapted for this study, in addition to some investigator-created items developed in collaboration with refugee community members and linked to previous research. The full survey was translated and back translated from English to Rohingya, including several rounds of revisions prior to and following piloting.

### Measures

Measures focused on exposure to systematic human rights violations, traumatic events, daily stressors and mental health symptoms, including PTSD, depression, anxiety, and associated functional impairment.

#### Systematic human rights violations scale

A 23-item scale measuring systematic human rights violations against Rohingya communities in Myanmar was developed by researchers.^1^ This measure was based on information from focus groups and key informants, including Rohingya community leaders, and was cross-referenced with existing reports regarding human rights violations occurring in Rakhine State, Myanmar [17]. Respondents were instructed to answer questions based on the experience of Rohingya people in Rakhine State in the last six years. The scale is intended to measure individual perceptions of the experiences of Rohingya communities in Rakhine State as a whole. Although Rohingya have experienced persecution in Myanmar for generations, the timeframe of six years was selected to capture increasingly rigid restrictions beginning in 2012. This was also based on an assumption that memories for relatively recent events would be more accurate than reports of restrictions occurring many years earlier. Individual items referred to specific restrictions in Rakhine, yoked to examples (e.g., “were Rohingya people in Rakhine State blocked from travelling freely, for example, not being able to travel from one township to another without authorization or permission?”). Response options ranged from 1 (not at all) to 4 (extremely). Individual items included restrictions on: citizenship, documentation, voting, using the name ‘Rohingya’, religious practices, travel, education, working, holding government positions, accessing medical services, accessing legal services, meeting in groups, marriage, childbirth, building/repairing homes, expressing feelings/thoughts, pressure to accept unwanted documentation, and ‘not receiving the same protection and rights as others’. Cronbach’s ⍺ indicated acceptable internal consistency (.74). As a result, human rights violations, with the exception of “receiving the same rights as others” and “protected by security forces” were combined to create a sum score of systematic human rights violations.^2^

#### Trauma events inventory

A 38-item Trauma Events Inventory, previously used with Rohingya refugees and based on the Harvard Trauma Questionnaire (HTQ) was adapted for use in this study [9, 18]. The adaptation process included the addition of a few items based on literature review and focus group discussions. The new items reflected specific events that some Rohingya experienced during the most recent wave of violence, for example witnessing the destruction/burning of villages. Respondents were asked to indicate if they had “experienced any of the following events” during their lifetime. They were asked to indicate “yes” or “no” for each item, and to share whether the event took place in Myanmar, Bangladesh, or both. In contrast to the human rights violations scale, participants were asked to endorse items that they experienced directly. A total sum score of trauma events was calculated based on responses.^3^

#### Daily stressors

This scale includes 25 items measuring daily stressors in Bangladesh in the last month and (ever) previously in Myanmar. Most items were taken from the Humanitarian Emergency Settings Perceived Needs Scale (HESPER) [19]. Two items about harassment by security forces and the local population were also added based on piloting and focus group discussion. Participants were asked if they had a serious problem with... (food, water, shelter, etc.). Response options included “yes” or “no.” The 12 stressors included food, water, shelter, sanitation facilities, income, physical health, safety, education, fair access to aid, travel, harassment by police, and harassment by locals. A total environmental stressors score was calculated using the sum of the number of stressors endorsed.

#### PTSD scale

This scale includes 16 items from the PTSD symptom subscale of the Harvard Trauma Questionnaire (HTQ) [18]. The HTQ was developed for and has been widely used with other refugee populations, including other refugee populations from Myanmar [20, 21]. Participants were asked how much these symptoms had bothered them in the previous week, with response options ranging from 1 (not at all) to 4 (extremely). Cronbach’s ⍺ indicated good internal consistency (.94). A total symptom severity score was calculated by averaging all items in the scale for each participant.

#### Depression and anxiety scales

This scale includes 29 items, 25 of these are from the anxiety and depression scales of the 25 item Hopkins Symptoms Checklist (HSCL-25), including ten anxiety symptom items and 15 depression symptom items [18]. To ensure that data was captured related to local expressions of distress associated with violence and displacement, a series of focus groups were conducted with the Rohingya population to investigate potential local expressions of distress. Based on feedback from these focus groups, an additional four items were developed by investigators: “bodily pain from distress/tension,” “feeling humiliated/subhuman,” “feeling disrespected,” and “feeling helpless.” The bodily pain item was included in the anxiety scale, but the remainder of the investigator-created items were examined individually. One item regarding suicidal ideation, “thoughts of ending life,” was removed from analysis due to apparent interviewer effects.^4^ Response options for all items ranged from 1 (not at all) to 4 (extremely). Cronbach’s ⍺ indicated good internal consistency for both depression (.92) and emotional distress (.96) scales. An emotional distress symptom score was calculated by averaging depression and anxiety items.

#### Functioning

This investigator-developed scale includes a total of five items. Four items focus on difficulties in daily functioning in the previous two weeks, and one item focuses on respondent perception of the reason for the difficulties (“physical health,” “mental health,” “current living situation,” or “other” with an option to explain; more than one response option could be selected). Four items were created following focus group feedback about typical daily tasks. Two of these items are typically gender specific, so separate examples were created for men and women. Response options for all items ranged from 1 (not at all) to 4 (extremely).

#### Analysis

5 Data analysis was conducted using IBM SPSS software. Mean imputation was used to address missing data, in order to retain power and avoid potential bias that can occur with list-wise deletion of data [22]. Mean imputation was used on relatively few items, on average less than one item per survey (0.65). In cases where large amounts of data were missing (an entire scale, or several items on a scale), no mean imputation was used, instead the entire scale in question was excluded from analysis. Analysis of the data was conducted both with and without mean imputation. Mean imputation did not change the direction of any of the primary findings, A few extreme outliers (more than 3.0 standard deviations from the mean) were Winsorized to the nearest score within 3.0 SD above/below the mean [23]. These adjustments did not change the pattern of any of the primary findings, so results are reported with outliers modified.

For open-ended qualitative survey responses two coders worked with the data. Two rounds of reviews of responses were conducted, resulting in a set of standardized categories. Qualitative responses were then sorted into these categories by the two coders and analyzed. Responses that were unclear or unintelligible were categorized as unspecified “other.” Following the completion of the coding, thematic categories were analyzed for frequency.

Specific regression models were investigated based on theoretical frameworks linked to previous research with Rohingya, in addition to focus group discussions and key informant interviews, and taking into account initial correlations between variables of interest. Models examined predictors of mental health outcomes, and functioning. In determining the most parsimonious set of final ‘predictor’ variables in each model, typically only those with a beta greater than .1 were included. The exception to this was for some variables that have a strong theoretical basis for inclusion (e.g., trauma history and PTSD symptoms).

## Results

The results presented describe systematic human rights violations, traumatic events, daily stressors, mental health symptoms, and functional impairment, and examine the associations between these factors.

### Demographics

Of the total households selected for inclusion, 168 (34%) were either not home or did not have an eligible respondent to complete the survey (often due to being a minor headed household, or because eligible respondents were not at home). In addition, 13 eligible respondents declined to participate in the survey. The final sample of 495 participants included 264 women (53%) and 231 men (47%), which closely matches the camp population gender breakdown of 56% women and 44% men. For more demographic data, see Additional File 1.

### Systematic human Rights violations

Response options for systematic human rights violations were, 1 = “Not at all”, 2 = “A little”, 3 = “Quite a bit”, and 4 = “Extremely.” Respondents endorsed restrictions against the Rohingya at the following rates: “blocked from. ..” **obtaining citizenship** (M = 3.99; 100% endorsed quite a bit or extremely), **working in government positions** (M = 3.99; 100% endorsed quite a bit or extremely**), obtaining official documentation** (M = 3.99; 99.8% endorsed quite a bit or extremely), **using the name Rohingya** (M = 3.98; 99.6% endorsed quite a bit or extremely), **expressing thoughts and feelings publicly** (M = 3.98; 99.8% endorsed quite a bit or extremely), **meeting in groups in public** (3.97; 99.6% endorsed quite a bit or extremely), **travelling** (3.96; 99.6% endorsed quite a bit or extremely), **religious practices** (3.96; 99.6% endorsed quite a bit or extremely), **voting** (3.96; 99.2% endorsed quite a bit or extremely), **legal services** (3.95; 100% endorsed quite a bit or extremely), **pressure to accept unwanted documentation** (3.95; 99.8% endorsed quite a bit or extremely), **restrictions in building or repairing homes** (3.90; 99.6% endorsed quite a bit or extremely), **pursuing education** (3.90; 99.6% endorsed quite a bit or extremely), **restrictions related to marriage** (3.81; 99.6% endorsed quite a bit or extremely), **medical services** (3.80; 99.6% endorsed quite a bit or extremely), **working** (3.78; 98.8% endorsed quite a bit or extremely), and **having children** (3.65; 98.8% endorsed quite a bit or extremely). For qualitative interpretative data related to these results, see Additional File 2 (Table 1).

**Table 4:** Percentage of respondents reaching diagnostic cutoff scores

Scale	Mental health composite score threshold	%
**PTSD**	Respondents who scored higher than the typically diagnostic cutoff score of 2.5	61.2%
**Emotional Distress (Anxiety and Depression)**	Respondents who scored higher than the typically diagnostic cutoff score of 1.75	84.0%

### Trauma events

Rohingya refugees endorsed a variety of potentially traumatic events *occurring in Myanmar*. The average number of trauma events endorsed for Myanmar was 19.4. The most frequently endorsed events related to exposure to violence, and included “exposure to frequent gunfire” (98.6%), “witnessed destruction burning of villages” (97.8%), “witnessed dead bodies” (91.8%), and “witnessed physical violence against others” (90.4%). Other events were related to directly experienced physical violence, including torture (55.5%), being beaten (46.1%), stabbed (29.4%), shot (5.1%), etc. Experiences of sexual assault were endorsed by both men and women. Women (33.1%) and men (34.3%) endorsed sexual assault at very similar rates. However, rape (by both security forces and others) was endorsed at a higher rate by women (3.1%) than men (0.8%). For qualitative interpretative data related to these trauma events results, see Additional File 2, and for a list of trauma events disaggregated by gender, see Additional File 3 (Table 2).

**Table 5:** Models predicting PTSD, emotional distress, and functioning difficulty

Variables	Model 1: Predicting PTSD symptoms R^**2**^ = .583, F (8, 469) = 82.05 p < .001	Model 2: Predicting emotional distress (anxiety and depression) R^**2**^ = .382, F (6, 471) = 48.47 p < .001	Model 3: Predicting functioning difficulty R^**2**^ = .451, F (6, 483) = 66.26 p < .001
**1. Systematic human rights violations**	Stand. β = .095	Stand. β = .160	Variable not included in this model
	*t =* 2.756	*t =* 3.890	
	*p =* .006**	*p =* .000**	
**2. Trauma history**	Stand. β = .185	Stand. β = .341	Stand. β = .033
	*t* = 5.417	*t =* 8.461	*t =* .834
	*p* = .000**	*p =* .000**	*p =* .405
**3. Bangladesh daily stressors**	Stand. β = .000	Stand. β = .105	Stand. β = .336
	*t* = .007	*t =* 2.725	*t =* 9.258
	*p* = .994	*p =* .007**	*p =* .000**
**4. Myanmar daily stressors**	Stand. β = .000	Stand. β = .337	Variable not included in this model
	*t* = 2.461	*t =* 9.029	
	*p* = .014*	*p =* .000**	
**5. Sex**	Stand. β **=** −.094	Stand. β = .037	Stand. β = −.065
	*t* = − 2.488	*t =* .868	*t =* − 1.850
	*p* = .013*	*p =* .386	*p =* .065
**6. Age**	Stand. β **=** .097	Stand. β = .109	Stand. β = −.017
	*t* = 3.147	*t =* 2.911	*t =* −.471
	*p* = .002**	*p =* .004**	*p =* .638
**7. Feeling humiliated/subhuman**	Stand. β = .313	Variable not included in this model	Variable not included in this model
	*t* = 6.885		
	*p* = .000**		
**8. Feeling helpless**	Stand. β = .366	Variable not included in this model	Variable not included in this model
	*t* = 8.807		
	*p* = .000**		
**9. PTSD symptoms**	Variable not included in this model	Variable not included in this model	Stand. β = .140
			*t* = 1.996
			*p* = .047*
**10. Depression symptoms**	Variable not included in this model	Variable not included in this model	Stand. β = .362
			*t* = 4.939
			*p* = .000**

*p < .05, **p < .01

### Daily stressors

Rohingya refugee respondents reported varying levels of daily stressors during the last month in Bangladesh and previously in Myanmar. In Bangladesh the most frequently endorsed stressors were regarding difficulties with sufficient income (95%), food (79%), limited access to education (72%), and travel (66%). Problems with living space (62%), sanitation facilities (62%), physical health (62%), and water (60%), were other problems endorsed by a majority of participants linked to current life in the camps in Bangladesh. Regarding stressors during their previous time living in Myanmar, the following was most commonly reported: serious problems - because of harassment by police (98%), harassment by the local population (97%), with travel (96%), and with education (84%). The average number of daily stressors endorsed by participants previously in Myanmar was 6.17 while currently in Bangladesh the average number of daily stressors endorsed was 6.34, although notably, the type of stressors differed across the two contexts (Table 3).

**Table 1:** Systematic human rights violations by severity

	“Were Rohingya people in Rakhine State blocked/prevented from.. .	Average Score
1	Obtaining **citizenship** (for example were Rohingya people blocked from have the same citizenship status as other ethnic groups in Rakhine State)	**3.99**
2	**Working in government positions**	**3.99**
3	Obtaining official **documentation** (such as National Registration Card (NRC), etc.)	**3.99**
4	**Using the name Rohingya** (for example at work, school, or in front of officials, etc.)	**3.98**
5	**Expressing thoughts** and feelings (for example publicly expressing desire for changes in Rakhine State, freely speaking to the press about the situation in Rakhine, etc.)	**3.98**
6	**Meeting in groups** in public	**3.97**
7	**Travelling** (for example not being able to travel from one township to another without authorization or permission)	**3.96**
8	**Religious practices** (for example going to musjid, madrassa, burial rituals, call to prayer, etc.)	**3.96**
9	**Voting**	**3.96**
10	**Legal Services** (for example access to legal defense, court systems, etc.)	**3.95**
11	**…** Pressured to accept **unwanted documentation** (for example National Verification Card (NVC), or other unwanted documentation)	**3.95**
12	**Building or repairing houses**	**3.90**
13	Pursuing **education (**for example blocked from attending government schools, universities, or blocked from pursuing chosen field of study)	**3.90**
14	**Marriage** (for example by being denied authorization to marry by authorities, or charged large amounts of money for permission to marry by authorities)	**3.81**
15	**Medical Services** (for example being refused care at a medical facility, or being prevented from travelling to a medical facility for care?)	**3.80**
16	**Working** (for example prevented from accessing fields, fishing boats, etc., or prevented from going to work)	**3.78**
17	**Having Children** (for example because of restrictions on family size, difficulties legally registering new births, etc.)?	**3.65**
18	**… Protected by security forces** (for example, protected against violence from Rakhine people)	**1.14**
19	**… Given same rights** as other ethnic groups (for example did Rohingya people have the same rights and privileges as Rakhine people, Burmese people, and other ethnic groups)	**1.13**

Response options: 1 = “Not at all”, 2 = “A little”, 3 = “Quite a bit”, and 4 = “Extremely”

### Posttraumatic stress symptoms

On a scale from 1 to 4 (1 = not at all, 2 = a little, 3 = quite a bit, 4 = extremely), all PTSD symptoms were endorsed at an average severity score of 2.5 or higher. The items with the highest average severity scores included “recurrent thoughts or memories of the most hurtful or terrifying events” (3.56), “feeling as though the event is happening again” (3.42), “feeling as if [they] don’t have a future” (2.91), and “recurrent nightmares” (2.83). Although the PTSD subscale of the HTQ has not been validated for use with the Rohingya population, a composite cut-off score has typically been used to indicate scores that are diagnostic of PTSD [24], **61.2% of participants endorsed posttraumatic stress symptoms typically diagnostic of PTSD**, with the average score for all participants being 2.80. For endorsement rates of all PTSD symptoms, see Additional File 4, and for more information related to the scoring and interpretation of the HTQ, see Additional File 5.

### Depression and anxiety symptoms

On a scale from 1 to 4 (1 = not at all, 2 = a little, 3 = quite a bit, 4 = extremely), all anxiety and depression symptoms were endorsed at an average severity score of 2.0 or higher. The anxiety and depression symptoms with the highest average severity scores included “worrying too much about things” (3.49), “feeling sad” (3.40), and “loss of interest in things you previously enjoyed doing” (3.04). Anxiety symptoms with the highest average severity scores included “feeling tense or keyed up” (3.13), “faintness, dizziness, or weakness” (2.73), and “headaches” (2.57). Investigator-developed items were endorsed at the following rates, “feeling humiliated/subhuman” (2.69), “bodily pain from distress/tension” (2.66),^6^ “feeling disrespected” (2.54), and “feeling helpless” (2.47). For qualitative interpretative data related to “feeling humiliated/subhuman”, see Additional File 2. Although the HSCL-25 has not been validated for the Rohingya population, a composite cut-off score for the combined anxiety and depression sub-scales has typically been used to indicate scores that are “checklist positive for some type of unspecified emotional distress” related to anxiety and depression [24],^7^**84.0% of respondents endorsed anxiety and depression symptoms typically indicative of emotional distress**, with average score for all participants being 2.64. For endorsement rates of all anxiety and depression symptoms, see Additional File 4. For more information related to the scoring and interpretation of the HSCL-25, see Additional File 5 (Table 4).

**Table 2:** Trauma events

	Myanmar	Bangladesh
Average number of trauma events experienced by Rohingya refugees in Bangladesh and Myanmar	**19.4**	**1.0**
**Trauma event endorsement rate**		
Exposure (i.e., hearing and/or seeing) to frequent gunfire	**98.6%**	**1.6%**
Witnessed destruction/burning of villages	**97.8%**	**2.0%**
Repeatedly exposed to violent images against Rohingya on websites (i.e., Facebook, RVision, TV, WhatsApp, etc.)	**95.3%**	**88.7%**
Forced to do things against religion (e.g., eat pork, remove cap/niqab/veil, burn/cut beard, etc.)	**94.9%**	**0.0%**
Threats against your ethnic group	**93.3%**	**0.6%**
Home destroyed	**93.1%**	**0.6%**
Witnessed dead bodies	**91.8%**	**2.8%**
Witnessed physical violence against others	**90.4%**	**1.4%**
Confiscation/looting of personal property	**88.2%**	**1.2%**
Murder of extended family or friend	**86.2%**	**0.2%**
*Follow-up to above item: Family member was killed by security forces	***100.0%***	***N/A***
Threats against you or your family	**83.7%**	**1.6%**
Forced to flee under dangerous conditions	**83.7%**	**0.4%**
Extortion (i.e., paying money due to force or threats)	**83.1%**	**2.8%**
Forced to hide because of dangerous conditions	**75.5%**	**1.0%**
Death of family or friends while fleeing or hiding (e.g., not from violent injury like shooting or stabbing, but because of illness, lack of food, drowning etc.)	**70.6%**	**2.0%**
Witnessed sexual violence/abuse of others	**67.3%**	**0.8%**
Unjust detainment	**63.3%**	**1.4%**
Present while security forces forcibly searched for people or things in your home (or the place where you were living)	**56.9%**	**1.2%**
Torture (i.e., while in captivity you received deliberate and systematic infliction of physical or mental suffering)	**55.5%**	**1.4%**
Forced labor (i.e., forced to do work that you could not decline, for example, patrolling, working for security forces, etc.)	**48.6%**	**0.2%**
Beaten by non-family member	**46.1%**	**1.6%**
Turned back while trying to flee	**46.1%**	**0.2%**
Sexual abuse, sexual humiliation, or sexual exploitation (e.g., coerced sexual acts, inappropriate touching, forced to remove clothing, etc.)	**33.3%**	**1.0%**
Murder of immediate family member (i.e., father, mother, sister, brother, husband/wife, or children)	**29.5%**	**0.0%**
*Follow-up to above item: Family member was killed by security forces	***99.3%***	***N/A***
Physical injury from being intentionally stabbed or cut with object (e.g., knife, axe, sword, machete, etc.)	**29.4%**	**1.8%**
Disappearance of family member	**19%**	**0.2%**
Beaten by spouse or family member	**14.5%**	**3.0%**
Other serious physical injury from violence (e.g., shrapnel, burn, landmine injury, etc.)	**9.2%**	**0.2%**
Forced Abortion (only female)	**5.4% (of female respondents)**	**0.0%**
Physical Injury from being shot (bullet wound)	**5.1%**	**0.2%**
Rape by security forces (i.e., forced to have unwanted sexual relations with security forces)^g^	**1.6%**	**0.0%**
Rape by others (i.e., forced to have unwanted sexual relations with a stranger, acquaintance, or family member)	**1.2%**	**0.0%**

### Functioning

Participants endorsed difficulties with daily functioning on a scale from 1 to 4 (1 = not at all, 2 = a little, 3 = quite a bit, 4 = extremely). Participants on average indicated difficulty carrying out daily tasks (2.87), caring for their hygiene (2.67), and engaging in social activities (2.39). There was a much lower level of difficulty in engaging in religious activities (1.60). For participants that indicated any level of functional difficulty, a follow-up item inquired “What do you attribute these difficulties to?” Respondents were instructed that they could choose more than one response and were given four response options including “current living situation” (71.6%), “mental health” (62.3%), “physical health” (48.2%), and “Other (Specify).” Of the specified qualitative responses, the most common reasons given were related to lack of income/opportunity (5.9%), displacement/statelessness (1.8%), and monsoon season (1.5%). For full text of functioning difficult items and endorsement rates, see Additional File 6: Functioning Difficulty Items.

### Prediction models

A series of initial multiple linear regression models were conducted in order to identify the most robust predictors for the final regression models. Generally, predictors were chosen that exceeded a β cutoff of .1; however, some variables with less than a β of .1 were included based on their broadly documented relationship with outcome variables, as well as their clinical and cultural significance in relation to outcome variables.

The three final models, presented here, predict –

- PTSD symptoms,
- emotional distress (anxiety and depression), and
- functioning^8^

As a reminder, the variable ‘Myanmar systematic human rights violations’ is a sum score that combines most of the items on the systematic human rights violations scale. ‘Trauma history’ is a sum score that combines the lifetime trauma events endorsed by a respondent in both Myanmar and Bangladesh, although nearly all events endorsed occurred in Myanmar. ‘Bangladesh daily stressors’ is a sum score that combines all the daily stressors endorsed in Bangladesh in the last month, while ‘Myanmar daily stressors’ is a sum score of the same stressors, except faced when the participants previously lived in Myanmar. ‘Depression symptoms’ is the composite score of HSCL depression items.

#### PTSD symptoms

The final model predicting PTSD symptoms included age, sex/gender, Bangladesh daily stressors, Myanmar daily stressors, trauma history, Myanmar systematic human rights violations, feeling humiliated/subhuman, and feeling helpless.^9^ The full regression model was significant in predicting PTSD scores *F*(8, 469) = 82.05; *p* < .001, and R^2^_=_ .58. Older age (β = .097, *p* < .01), being a woman (β = −.094, *p* < .05), a higher number of lifetime trauma events (β = .185, p < .001), higher levels of systematic human rights violations in Myanmar (β = .095, p < .01), a higher number of daily stressors in Myanmar (β = .000, p < .05), higher levels of feeling humiliated/subhuman (β = .313, *p* < .001), and higher levels of feeling helpless (β = .366, *p* < .001), significantly predicted higher PTSD scores.

#### Emotional distress (anxiety and depression)

The final model predicting emotional distress (anxiety and depression) symptoms included age, sex/gender, Bangladesh daily stressors, Myanmar daily stressors, trauma history, and Myanmar systematic human rights violations. The full model was significant in predicting distress scores *F*(6, 471) = 48.47; *p* < .001, and R^2^_=_ .38. Older age (β = .109, *p* < .01), a higher levels of daily stressors in Bangladesh (β = .105, p < .01), higher levels of daily stressors previously in Myanmar (β = .337, *p* < .001), a higher number of lifetime trauma events (β = .341, *p* < .001), and higher levels of systematic human rights violations in Myanmar (β = .160, p < .001) significantly predicted higher emotional distress scores.

#### Functioning difficulties

The final model predicting functioning difficulties included the following predictor variables, age, sex/gender, Bangladesh daily stressors, trauma history, PTSD symptoms, and depression symptoms. The full regression model was significant in predicting functioning difficulties *F*(6, 483) = 66.26; *p* < .001, and R^2^_=_ .45. Higher numbers of Bangladesh daily stressors (β = .336, p < .001), higher levels of depression symptoms (β = .362, p < .001), and higher levels of PTSD symptoms (β = .140, *p* < .05) significantly predicted higher levels of functioning difficulty (Table 5).

**Table 3:** Daily stressors in Bangladesh and Myanmar

Bangladesh Daily Stress: *“****During the past month*** *have you had a serious problem.*. *.”*	%	Myanmar Daily Stress: “*In Myanmar did you* ***generally have a*** *serious problem.*. *.”*	%
“Because you do not have enough **income**, money, or resources to live”	95%	“Because you do not have enough **income**, money, or resources to live”	30%
“**Food**, for example, because you do not have enough food, or good enough food, or because you are not able to cook food”	79%	“**Food**, for example, because you do not have enough food, or good enough food, or because you are not able to cook food”	24%
“Because your family are not in **school**, or are not getting a good enough education”	72%	“Because your family are not in **school**, or are not getting a good enough education”	84%
“**Move between places**, for example, problems with travel due to checkpoints, extortion, being turned back while trying to travel to a place, etc.”	66%	“**Move between places**, for example, problems with travel due to checkpoints, extortion, being turned back while trying to travel to a place, etc.”	96%
**“Suitable place to live in**, for example because of inadequate shelters or amount of space”	62%	**“Suitable place to live in**, for example because of inadequate shelters or amount of space”	7%
“Safe access to clean toilet and **sanitation facilities”**	62%	“Safe access to clean toilet and **sanitation facilities”**	11%
“**Physical health**, for example, because you have a physical illness, injury, or disability”	62%	“**Physical health**, for example, because you have a physical illness, injury, or disability”	42%
**“Water** that is safe for drinking or cooking”	60%	**“Water** that is safe for drinking or cooking”	17%
“**Fair access** to the aid that is available from agencies working in the area”	47%	“**Fair access** to the aid that is available from agencies working in the area”	44%
**“Not safe** or protected where you live now, for example, because of conflict, violence or crime in your community”	14%	**“Not safe** or protected where you lived, for example, because of conflict, violence or crime in your community”	66%
**“Harassment by the local population**, for example being threatened, insulted, or extorted, etc.”	13%	“**Harassment by the local population**, for example being threatened, insulted, or extorted, by Rakhine, Hindu, or Dinet, etc.”	97%
**“Harassment by police** or security forces, for example being threatened, insulted, or extorted, etc.”	4%	**“Harassment by police** or security forces, for example being threatened, insulted, or extorted, etc.”	98%

For additional data from final regression models, see Additional File 7.

## Discussion

Respondents reported high levels of systematic human rights violations in Rakhine State in recent years (most respondents responded with “quite a bit” to “extremely” for each of the human rights violation-related items, focused on the period from 2012 on). High levels of physical violence in Myanmar were reported including torture, being beaten, stabbed, shot, and/or sexually assaulted. High levels of current daily stressors in the camps in Bangladesh were also reported including lack of adequate income, insufficient food, limited access to education, and limited freedom of movement. Symptoms of mental health distress were high in this study. Using reference cutoff scores, rates of PTSD and emotional distress (anxiety and depression) were 61.2 and 84.0% respectively. Additionally, 79.2% of respondents indicated experiencing some level of “bodily pain from distress/tension,” and 68.7% of respondents reported currently feeling “humiliated or subhuman.” In regression models, trauma history and systematic human rights violations significantly predicted PTSD and emotional distress (anxiety and depression symptoms). Mental health symptoms (PTSD and depression) as well as daily stressors encountered in camps in Bangladesh significantly predicted difficulties in functioning.

This study contributes to this growing body of literature examining the impact of traumatic and daily stressors on mental health outcomes. In addition, this study extends this literature, by exploring the potential impact of historical systematic human rights violations on mental health outcomes.

### Systematic human rights violations

Human rights violations in Myanmar and against the Rohingya have been documented in human rights reports [1, 25]. However, the few formal studies focusing on human rights violations against the Rohingya in Myanmar use nonrepresentative sampling methods, rely on qualitative information, and generally focus on a small subset of human rights violations linked to specific incidents of violence [6, 26, 27]. One qualitative study conducted with Rohingya village leaders highlighted severe restrictions on travel, marriage, education, legal rights and denial of citizenship rights in Myanmar [27]. This study builds on these efforts by utilizing a representative sample of Rohingya refugees residing in the camps in Bangladesh, and by examining an expansive list of human rights violations, revealing a history of systematic human rights violations in Myanmar. Such systematic human rights violations prevented them from exercising their rights in many areas of life, including restrictions on travel, livelihoods, housing, education, expression of cultural identity, family planning, social, religious, and political life.

### Trauma events

High levels of physical violence in Myanmar were reported by the Rohingya participants of this study. Medecins Sans Frontieres (MSF) estimates that at least 6700 Rohingya were killed in the violence during August/September of 2017 [28]. Nearly all respondents indicated horrific experiences in Myanmar, including 98.6% exposed to frequent gunfire, 97.8% witnessed the destruction/burning of villages, 91.8% saw dead bodies, and 90.4% witnessed physical violence against others. When compared to a cross-sectional study conducted in 2013 with 148 long-standing registered Rohingya refugees in Bangladesh, this study with 495 recent arrivals (average time in Bangladesh = 18 months) indicated that recent arrivals have experienced higher rates of physical violence, sexual assault, and more [9]. Additionally, 95.3% indicated that they were “repeatedly exposed to violent images against Rohingya on websites,” which highlights the potential for retraumatization of refugees due to repeated online exposure to violent images, as has been the case in other studies with refugees and exposure to media related to violence [29].

Comparing the levels of trauma exposure in Bangladesh and Myanmar is illuminating. On average, respondents endorsed 19 potentially traumatic events in Myanmar, and on average, only 1 potentially traumatic event in Bangladesh (the most common being exposure to violent images against Rohingya on websites). Although most respondents have spent far more years in Myanmar than Bangladesh, it is unlikely that the difference in time alone accounts for the discrepancy in exposure to these events.

### Daily stressors

High levels of current daily stressors such as lack of adequate income, insufficient food, and limited access to education were reported by Rohingya refugees. These findings closely mirror findings from previous studies with registered Rohingya refugees in Bangladesh, and emphasize the difficult living conditions for Rohingya refugees [9]. In contrast to Bangladesh, where several of the primary concerns are over basic needs, the highest endorsed daily stressors in Myanmar were serious problems due to harassment by police, harassment by the local population, problems with travel, and limited access to education.

### Mental health distress

Symptoms of mental health distress were high in this study. The World Health Organization (WHO)/UNHCR toolkit guidance estimates that prior to an emergency 10% of an adult population will be affected by some type of moderate or mild mental health disorder, this rate is expected to increase by 5–10% to a total of 15–20% in adult populations affected by emergencies, 12 months after an emergency (including mild and moderate depression and anxiety, and mild and moderate PTSD) [19]. However, cutoff scores documenting rates for PTSD (61.2%), and emotional distress (anxiety and depression (84.0%)) in this study were much higher than these expected percentages.^10^ Cutoff scores for PTSD also far surpassed those of a previous study in 2013 with registered Rohingya refugees living in the camps in Bangladesh for many years, using similar measures. Results from that study found that 36% of long-standing registered Rohingya refugees met the cutoff score for PTSD, while depression scores from the same study were similar to those measured in this study at 89% [9]. The elevated PTSD scores in this study could be due to a number of factors including the number and severity of traumatic events and human rights violations, as well as the recency of these experiences compared to those in the previous study. Although the number of participants that met cutoff scores for PTSD is higher than expected, these rates are similar to other conflict-affected refugee or displaced populations. Such as Syrian refugees in Kurdistan (59.4%), and internally displaced persons in Uganda (54%) [30, 31].

Intrusive trauma symptoms, typically associated with PTSD were notably the most highly endorsed trauma symptoms. On the scale 1 = not at all, 2 = a little bit, 3 = quite a bit, 4 = extremely, “recurrent thoughts or memories of the most hurtful or terrifying events” (3.56), “feeling as though the event is happening again” (3.42), and “recurrent nightmares” (2.83) were notably the most highly endorsed trauma symptoms. While “worrying too much about things” (3.49), “feeling sad” (3.40), and “feeling tense or keyed up” (3.13) were also highly endorsed on anxiety and depression scales.

Investigator created items that were designed based on Rohingya feedback are also an important means of understanding mental health distress. Psychosomatic pain was often brought up spontaneously by focus group respondents when discussing the impact of violence and displacement on the Rohingya, and 79.2% of respondents indicated experiencing some level of “bodily pain from distress/tension,” congruent with a previous study of Rohingya refugees, but much higher than a more recent humanitarian assessment using a non-random sample that reported 20% of Rohingya refugee respondents endorsed “somatic complaints” [9, 10]. The last investigator created item, feeling “humiliated or subhuman” was endorsed by 68.7% of respondents, highlighting feelings of dehumanization likely resulting from systematic human rights violations and inadequate living conditions in the refugee camps.

### Prediction models

The long-term implications of systematic human rights violations, traumatic experiences, and daily stressors, are emphasized when examining the significant role such factors play in predicting mental health outcomes and functioning.

In regression models, as expected, trauma history significantly predicted PTSD and emotional distress (anxiety and depression symptoms). However, in addition to trauma history and daily stressors encountered in Myanmar, systematic human rights violations in Myanmar also predicted both PTSD and emotional distress (anxiety/depression). While the impact on mental health of trauma exposure, and to a lesser extent daily stressors, has been studied, there is a lack of information on how systematic human rights violations may impact mental health outcomes [9, 15, 16]. This study emphasizes the importance of considering exposure to systematic human rights violations (e.g., being blocked/restricted from meeting in groups, participating in religious practices, marriage, childbirth, the ability to travel around the country, to access education and medical facilities) in predicting mental health outcomes.

While it was expected that trauma history, age (being older), and sex (being female) would significantly predict PTSD symptoms, which was the case, it was surprising that daily stressors encountered in Bangladesh did not significantly predict PTSD scores, particularly when the relationship between daily stressors associated with life in refugee camps in Bangladesh and PTSD symptoms has been documented in a previous study with the Rohingya [9]. This may be due to the relatively recent arrival of the majority of Rohingya refugees in this study compared to the previous study, which mostly included refugees who had lived in the refugee camps for several years. The shorter amount of time since arrival in the refugee camps, could mean that there has been less time to recover from events and conditions in Myanmar that contribute to mental health distress, and that these Myanmar events and conditions currently play a larger role in mental health outcomes. It may be that daily stressors encountered in the camps in Bangladesh will play a larger role in mental health symptoms the longer the Rohingya remain in the camps, as stress from these daily problems will likely accumulate over time. In contrast to daily stressors in Bangladesh, daily stressors experienced in Myanmar did predict PTSD symptoms, despite the fact that most of the newly arrived Rohingya refugees who participated in the study have lived in the Bangladesh camps for more than a year now. This could be interpreted as suggesting that immediately following a crisis, chronic and acute stressors happening prior to and around the time of the event may contribute more to PTSD symptoms than daily stressors in the months following the event. That having been said, emotional distress (anxiety and depression symptoms) were predicted by current stressors in Bangladesh as well as previous chronic daily stressors in Myanmar, so perhaps stressors in the months after an event play a larger role in influencing depression and anxiety symptoms than PTSD symptoms, especially in this particular population.

As expected, mental health symptoms (PTSD and depression) as well as daily stressors encountered in camps in Bangladesh significantly predicted difficulties in functioning (or ‘functional impairment’). The link between mental health symptoms and reduced functioning has been well-documented in refugee and other populations [32, 33]. The majority of respondents in this study (62.3%) attributed difficulties in daily functioning to their mental health symptoms. This finding suggests a recognition from Rohingya refugees regarding the extent to which their mental health challenges are affecting their everyday lives in terms of their ability to function.

### Limitations

This research has several limitations. First, the measures used to assess mental health symptoms have not been normed and validated for the Rohingya refugee population, thus rates of PTSD and emotional distress should be considered with caution. Of the households selected for inclusion, 34% were not home or did not have an eligible respondent, potentially introducing a level of non-response bias. Due to resource constraints, follow-up visits to selected households where participants were not available on the first visit was not possible. Interviews were conducted in the most private room of refugee shelters, however, shelters are constructed using bamboo and plastic sheeting, making it difficult to guarantee participant privacy. This could have impacted participants willingness to discuss sensitive issues. Finally, this data is cross-sectional as such, the direction of relationships between variables may not always be clear; longitudinal research is needed.

## Conclusion

The findings emphasize the importance of examining links between human rights violations and mental health, with an eye towards preventative strategies. Service providers working with humanitarian populations should continue to provide mental health services aimed at reducing PTSD and emotional distress (anxiety and depression), as well as strengthening coping strategies and resilience. However, the findings of this study suggest that addressing mental health symptoms alone is insufficient. Providers must address systematic human rights violations and daily stressors by advocating for the removal of systemic barriers to human rights and improving conditions in refugee camps to ensure that basic needs are met. Addressing systematic human rights violations will likely require significant resources for long-term advocacy efforts. Findings from this study are particularly salient for those working with Rohingya populations, but they are also more broadly relevant for other conflict-affected and persecuted communities where basic human rights are systematically violated.

## Supplementary information

**Additional file 1.** Full Demographic Data Table. Description of data: A table containing the demographic results from the participants in the study.

**Additional file 2.** Relevant Qualitative Data. Description of data: Following initial analyses of quantitative data, focus group discussions were held with Rohingya field researchers to investigate assumptions and assist in interpretation of findings. These discussions were recorded and transcribed. Examples of this feedback are provided here, to aid in interpretation of survey findings.

**Additional file 3.** Trauma Events Disaggregated by Gender. Description of data: Endorsement rates for all trauma events in both Myanmar and Bangladesh disaggregated by gender.

**Additional file 4.** Mental Health Symptom Endorsement Rates. Description of data: Endorsement rates for individual mental health symptoms, including PTSD, anxiety and depression symptoms, and investigator-developed items.

**Additional file 5.** Interpretation of Mental Health Scales. Description of data: Additional information regarding the scoring, interpretation, and limitations of the mental health measures used.

**Additional file 6.** Functioning Difficulty Items. Description of data: Full text of functioning items and associated average scores.

**Additional file 7.** Full Data from Final Regression Models. Description of data: Full data for the final regression models presented in the manuscript.

## Data Availability

The datasets used and analyzed for this study are available from the corresponding author on reasonable request.

## Abbreviations

HESPER: Humanitarian emergency settings perceived needs scale
HSCL-25: Hopkins symptoms checklist-25
HTQ: Harvard trauma questionnaire
INGO: International non-governmental organization
IOM: International Organization for Migration
IRB: Institutional review board
MHPSS: Mental health and psychosocial support
MSF: Medecins Sans Frontieres
NRC: National registration card
NVC: National verification card
PPS: Probability proportionate to size
PTSD: Post-traumatic stress disorder
SD: Standard deviation
SPSS: Statistical package for the social sciences
UNHCR: United nations high commissioner for refugees
WHO: World Health Organization

## Footnotes

1 Data collected on systematic human rights violations is self-report data, which implies that findings regarding systematic human rights violations presented in this report are based on Rohingya perceptions of human rights violations that their communities have experienced.

2 These items were the only reverse-coded items on this scale and were removed from the sum score due to concerns over respondent confusion and the internal consistency of the scale. However, removing these items did not change the pattern of any primary findings. These items are reported individually below, and the majority of participants answered in the direction that was expected.

3 During the analysis, one item was dropped from the trauma events scale due to apparent interviewer effects on this item.

4 One item regarding suicidal ideation, “thoughts of ending life,” was removed from analysis due to apparent interviewer effects. Because suicidal ideation has large cultural/religious implications for the Rohingya, there is a stigma to endorsing items regarding suicidal ideation. It seems that there was still a hesitancy by respondents to endorse suicidal ideation to all the field researchers except the one with the most mental health experience.

5 The datasets used and analyzed for this study are available from the corresponding author on reasonable request.

6 The bodily pain item was used as part of the anxiety sub-scale and total emotional distress score; however, the remainder of the investigator-created items were examined individually.

7 Unspecified emotional distress will be referred to in this report as “emotional distress,” and is a combination of anxiety and depression subscales from the HSCL-25.

8 The multicollinearity statistics were examined for all models, and all predictors had acceptable VIF scores < 5.

9 Feeling humiliated/subhuman and feeling helpless were items that were developed from discussions with Rohingya key informants and focus groups. These expressions of distress seemed to be distinctly different than items included in the mental health scales utilized in this study. Feeling humiliated/subhuman captures the feelings associated with being referred to as an animal or forced to survive in living situations not suited for human beings. Feeling helpless captures the feeling associated with the lack of empowerment or agency to be able to change or improve one’s situation. Although, during the course of the questionnaire refugees were asked if they felt this way in the last two weeks, it seemed that these feelings built-up over the course of a long period of time. Focus group participants reported feeling both humiliated/subhuman and helpless both historically in Myanmar and currently in their lives in Bangladesh. Results from the correlation matrix show that these items were both significantly correlated with PTSD scores, and were included as independent variables in the final regression model predicting PTSD symptoms.

10 These results should be used with caution. As mentioned in the limitations, this instrument has not been normed and validated for use with this population.
