# Supplementary material for: Systematic human rights violations, traumatic events, daily stressors and mental health of Rohingya refugees in Bangladesh
Source: Confl Health. 2020 Aug 20;14:60. doi: 10.1186/s13031-020-00306-9 (PMC7441657; doi:10.1186/s13031-020-00306-9)
Supplement: Supplementary file 1 — Additional file 1. Full Demographic Data Table. Description of data: A table containing the demographic results from the participants in the study. [file 13031_2020_306_MOESM1_ESM.docx]

**Title: Additional File 1: Full Demographic Data Table**

| Number of camp blocks sampled | 33 |
| --- | --- |
| Total sample size | 495 |
| **Household Inclusion** |  |
| Households selected for inclusion that were not able to participate | 168 |
| Households that declined to participate | 13 |
| **Gender** | **%** |
| Female | 53.3% |
| Male | 46.7% |
| **Age** | **Years** |
| Minimum | 18 years |
| Maximum | 75 years |
| Mean | 36 years |
| Median | 34 years |
| **Time since arrival in Bangladesh** | **Months** |
| Mean | 18 months |
| **Period of arrival** | **%** |
| Pre-October 2016 | 4.2% |
| Between October 2016 and August 2017 | 4.4% |
| Post August 2017 | 91.3% |
| **Country of birth** | ***N*** |
| Myanmar | 493 |
| Bangladesh | 2 |
| **Township of origin** | **%** |
| Maungdaw | 66.3% |
| Buthidaung | 28.4% |
| Rathedaung | 4.7% |
| Kyauktaw | 0.4% |
| Sittwe | 0.2% |
| **Education level completed** | **%** |
| Less than primary | 64.6% |
| Primary (1-4) | 12.3% |
| Secondary (5-8) | 4.6% |
| Tertiary (9-10) | 1.0% |
| University | 0.2% |
| Other/Religious education | 79% |
| **Religiosity (“How important are your religious beliefs to the way you live your life?”)** | **%** |
| Extremely | 99.2% |
| Quite a bit | 0.4% |
| A little | 0.0% |
| Not at all | 0.4% |
