# Supplementary material for: Systematic human rights violations, traumatic events, daily stressors and mental health of Rohingya refugees in Bangladesh
Source: Confl Health. 2020 Aug 20;14:60. doi: 10.1186/s13031-020-00306-9 (PMC7441657; doi:10.1186/s13031-020-00306-9)
Supplement: Supplementary file 2 — Additional file 2. Relevant Qualitative Data. Description of data: Following initial analyses of quantitative data, focus group discussions were held with Rohingya field researchers to investigate assumptions and assist in interpretation of findings. These discussions were recorded and transcribed. Examples of this feedback are provided here, to aid in interpretation of survey findings. [file 13031_2020_306_MOESM2_ESM.docx]

**Additional File 1: Relevant Qualitative Data**

Following initial analyses of quantitative data, focus group discussions were held with Rohingya field researchers to investigate assumptions and assist in interpretation of findings. These discussions were recorded and transcribed. Examples of this feedback are provided here, to aid in interpretation of survey findings.

**Focus group participants**

The eight field researchers participating in the focus group discussion ranged in age from 29 to 40 years. All could speak, read, and write in multiple languages, and all had prior experience working for I/NGOs in Rakhine State. The responses below were based on their experience conducting interviews during this study.

**Systematic human rights violations**

Restrictions include being ‘blocked from marriage’ primarily due to registration restrictions, extortion, and fear of arrest. One respondent stated that, *“Even if a person is over [the age of] 18, the immigration and village administration extort money for written permission for marriage.” “If you continue getting married [without permission] you could get arrested.”* Another stated, *“Many people didn’t get married because they couldn’t afford it, many people would flee to Bangladesh just to get married.”*

Another example is being ‘blocked from having children’ primarily due to the fear of children being “blacklisted.” *“Children get blacklisted if they are born outside of a registered marriage.” “Blacklisted children can’t do anything, they can’t attend school, they are not included on the official family list (official documentation that is needed for several different aspects of life in Rakhine), they can’t open a business or travel.”*

**Trauma events**

Respondents commented that torture is a common practice by security forces in Northern Rakhine and often includes physical abuse: *“[If] any Rohingya were arrested, they tortured [them] to get anything they wanted them to say, as well as to get money.” “The norm of being taken into custody for the Rohingya includes being beaten with a rod.”*

Respondents indicated that forced abortion involves women feeling forced to get an abortion due to violating the two-child policy enforced on Rohingya living in Rakhine, *“One woman knew that her family lists would be checked, and she was pregnant with her third child. She was afraid of being arrested and tortured, so she got an abortion.”* Respondents also indicated that this was related to the fear of having children that would be blacklisted and therefore barred from registration, education, livelihood and other opportunities.

Respondents indicated that violence of a religious nature in Rakhine included being “Forced to do things against religion” such as these examples:*“While a Rohingya cross[es] the check post on the way, Rohingya mullahs are forced to take off their caps from heads and women to take off their veils or niqab.”* Another respondent commented that *“Myanmar security forces always enter into the mosques and urinate inside, tear the Quran and other religious books.” “Rohingya people on pilgrimage are often abused physically and their beards are burnt after they are arrested.”*

**Feeling humiliated or subhuman**

An example of feeling “humiliated or subhuman” is exemplified by this quote: *“They call us like animals. When we are at school, they call the Muslim students “khoung” which means animal, not human being, also any activities regarding authorities that you want to do they will use the term khoung which means animals. . . when I was in class 6 or 7, I was 10 or 15 minutes late to school and the teacher said “de khoung” (animal) why are you so late today. It is a tool of discrimination, it makes me feel very bad. . .”*
