# Supplementary material for: Systematic human rights violations, traumatic events, daily stressors and mental health of Rohingya refugees in Bangladesh
Source: Confl Health. 2020 Aug 20;14:60. doi: 10.1186/s13031-020-00306-9 (PMC7441657; doi:10.1186/s13031-020-00306-9)
Supplement: Supplementary file 3 — Additional file 3. Trauma Events Disaggregated by Gender. Description of data: Endorsement rates for all trauma events in both Myanmar and Bangladesh disaggregated by gender. [file 13031_2020_306_MOESM3_ESM.docx]

**Additional File 3: Trauma Events Disaggregated by Gender**

| **Trauma events in Myanmar by sex** | **Female%** | **Male%** | **Total%** |
| --- | --- | --- | --- |
| Exposure (i.e. hearing and/or seeing) to frequent gunfire | 98.1% | 99.1% | **98.6%** |
| Witnessed destruction burning of villages | 97.7% | 97.8% | **97.8%** |
| Repeatedly exposed to violent images against Rohingya on websites (i.e. Facebook, RVision, TV, Whatsapp, etc.) | 96.2% | 94.3% | **95.3%** |
| Forced to do things against religion (e.g. eat pork, remove cap/ niqab /veil, burn/cut beard, etc.) | 94.7% | 95.2% | **94.9%** |
| Threats against your ethnic group | 97.3% | 88.7% | **93.3%** |
| Home destroyed | 92.4% | 93.9% | **93.1%** |
| Witnessed dead bodies | 89.6% | 94.3% | **91.8%** |
| Witnessed physical violence against others | 89.2% | 91.7% | **90.4%** |
| Confiscation/looting of personal property | 84.8% | 92.2% | **88.2%** |
| Murder of extended family or friend | 90% | 81.7% | **86.2%** |
| **Follow-up to above item: Family member was killed by security forces* | *100%* | *100%* | ***100.0%*** |
| Threats against you or your family | 95.4% | 70.4% | **83.7%** |
| Forced to flee under dangerous conditions | 76.3% | 92.2% | **83.7%** |
| Extortion (i.e. paying money due to force or threats) | 80.8% | 85.7% | **83.1%** |
| Forced to hide because of dangerous conditions | 75% | 76.1% | **75.5%** |
| Death of family or friends while fleeing or hiding (e.g., not from violent injury like shooting or stabbing, but because of illness, lack of food, drowning etc.) | 69.2% | 72.2% | **70.6%** |
| Witnessed sexual violence/abuse of others | 75.4% | 58.3% | **67.3%** |
| Unjust detainment | 52.7% | 75.2% | **63.3%** |
| Present while security forces forcibly searched for people or things in your home (or the place where you were living) | 53.8% | 60.4% | **56.9%** |
| Torture (i.e. while in captivity you received deliberate and systematic infliction of physical or mental suffering) | 40% | 73% | **55.5%** |
| Forced labor (i.e., forced to do work that you could not decline, for example, patrolling, working for security forces, etc.) | 14.2% | 87.4% | **48.6%** |
| Beaten by non-family member | 35% | 58.7% | **46.1%** |
| Turned back while trying to flee | 35% | 58.7% | **46.1%** |
| Sexual abuse, sexual humiliation, or sexual exploitation (e.g. coerced sexual acts, inappropriate touching, forced to remove clothing, etc.) | 33.1% | 34.3% | **33.3%** |
| Murder of immediate family member (i.e., father, mother, sister, brother, husband/wife, or children) | 41.4% | 16.4% | **29.5%** |
| **Follow-up to above item: Family member was killed by security forces* | *99.1%* | *100%* | ***99.3%*** |
| Physical injury from being intentionally stabbed or cut with object (e.g. knife, axe, sword, machete, etc.) | 17.3% | 43% | **29.4%** |
| Disappearance of family member | 28.5% | 8.3% | **19%** |
| Beaten by spouse or family member | 19.6% | 8.7% | **14.5%** |
| Other serious physical injury from violence (e.g., shrapnel, burn, landmine injury, etc.) | 4.6% | 14.3% | **9.2%** |
| Forced Abortion (Only female) | 5.4% | *N/A* | **5.4% (Only Female)** |
| Physical Injury from being shot (bullet wound) | 4.2% | 6.1% | **5.1%** |
| Rape by security forces (i.e. forced to have unwanted sexual relations with security forces) | 2.7% | 0.4% | **1.6%** |
| Rape by others (i.e. forced to have unwanted sexual relations with a stranger, acquaintance, or family member) | 1.5% | 0.9% | **1.2%** |

| **Trauma events in Bangladesh by sex** | **Female%** | **Male%** | **Total%** |
| --- | --- | --- | --- |
| Repeatedly exposed to violent images against Rohingya on websites (i.e. Facebook, RVision, TV, Whatsapp, etc.) | **88.3%** | **89.2%** | **88.7%** |
| Beaten by spouse or family member | **4.9%** | **0.9%** | **3.0%** |
| Extortion (i.e. paying money due to force or threats) | **2.3%** | **3.5%** | **2.8%** |
| Witnessed dead bodies | **2.3%** | **3.5%** | **2.8%** |
| Death of family or friends while fleeing or hiding (e.g., not from violent injury like shooting or stabbing, but because of illness, lack of food, drowning etc.) | **0.0%** | **4.3%** | **2.0%** |
| Witnessed destruction burning of villages | **3.0%** | **0.9%** | **2.0%** |
| Physical injury from being intentionally stabbed or cut with object (e.g. knife, axe, sword, machete, etc.) | **1.9%** | **1.7%** | **1.8%** |
| Exposure (i.e. hearing and/or seeing) to frequent gunfire | **0.8%** | **2.6%** | **1.6%** |
| Threats against you or your family | **1.1%** | **2.2%** | **1.6%** |
| Beaten by non-family member | **1.1%** | **2.2%** | **1.6%** |
| Torture (i.e. while in captivity you received deliberate and systematic infliction of physical or mental suffering) | **0.8%** | **2.2%** | **1.4%** |
| Unjust detainment | **0.8%** | **2.2%** | **1.4%** |
| Witnessed physical violence against others | **0.8%** | **2.2%** | **1.4%** |
| Confiscation/looting of personal property | **0.4%** | **2.2%** | **1.2%** |
| Present while security forces forcibly searched for people or things in your home (or the place where you were living) | **0.4%** | **2.2%** | **1.2%** |
| Forced to hide because of dangerous conditions | **0.0%** | **2.2%** | **1.0%** |
| Sexual abuse, sexual humiliation, or sexual exploitation (e.g. coerced sexual acts, inappropriate touching, forced to remove clothing, etc.) | **1.1%** | **0.9%** | **1.0%** |
| Witnessed sexual violence/abuse of others | **0.4%** | **1.3%** | **0.8%** |
| Threats against your ethnic group | **0.4%** | **0.9%** | **0.6%** |
| Home destroyed | **0.0%** | **1.3%** | **0.6%** |
| Forced to flee under dangerous conditions | **0.4%** | **0.4%** | **0.4%** |
| Disappearance of family member | **0.4%** | **0.0%** | **0.2%** |
| Other serious physical injury from violence (e.g., shrapnel, burn, landmine injury, etc.) | **0.4%** | **0.0%** | **0.2%** |
| Physical Injury from being shot (bullet wound) | **0.0%** | **0.4%** | **0.2%** |
| Forced labor (i.e., forced to do work that you could not decline, for example, patrolling, working for security forces, etc.) | **0.0%** | **0.4%** | **0.2%** |
| Turned back while trying to flee | **0.0%** | **0.4%** | **0.2%** |
| Murder of extended family or friend | **0.0%** | **0.4%** | **0.2%** |
| Forced to do things against religion (e.g. eat pork, remove cap/niqab/veil, burn/cut beard, etc.) | **0.0%** | **0.0%** | **0.0%** |
| Murder of immediate family member (i.e., father, mother, sister, brother, husband/wife, or children) | **0.0%** | **0.0%** | **0.0%** |
| Forced Abortion (Only female) | **0.0%** | **N/A** | **0.0% (Only Female)** |
| Rape by security forces (i.e. forced to have unwanted sexual relations with security forces) | **0.0%** | **0.0%** | **0.0%** |
| Rape by others (i.e. forced to have unwanted sexual relations with a stranger, acquaintance, or family member) | **0.0%** | **0.0%** | **0.0%** |
