# Supplementary material for: Systematic human rights violations, traumatic events, daily stressors and mental health of Rohingya refugees in Bangladesh
Source: Confl Health. 2020 Aug 20;14:60. doi: 10.1186/s13031-020-00306-9 (PMC7441657; doi:10.1186/s13031-020-00306-9)
Supplement: Supplementary file 4 — Additional file 4. Mental Health Symptom Endorsement Rates. Description of data: Endorsement rates for individual mental health symptoms, including PTSD, anxiety and depression symptoms, and investigator-developed items. [file 13031_2020_306_MOESM4_ESM.docx]

**Additional File 4: Mental Health Symptom Endorsement Rates**

**PTSD symptoms (1 = Not at all, 2 = A little, 3 = Quite a bit, 4 = Extremely)**

| # | **Symptom** | **Average Score** |
| --- | --- | --- |
| **1** | “Recurrent thoughts or memories of the most hurtful or terrifying events” | 3.56 |
| **2** | “Feeling as though the event is happening again” | 3.42 |
| **3** | “Feeling as if you don’t have a future” | 2.91 |
| **4** | “Recurrent nightmares” | 2.83 |
| **5** | “Feeling detached or withdrawn from people” | 2.83 |
| **6** | “Less interest in daily activities” | 2.82 |
| **7** | “Sudden emotional or physical reaction when reminded of the most hurtful or traumatic events, for example: sudden anxiety/stress or suddenly feeling heart racing, rapid breathing, etc.” | 2.82 |
| **8** | “Inability to remember parts of the most hurtful or traumatic events” | 2.78 |
| **9** | “Avoiding activities that remind you of the traumatic or hurtful event” | 2.74 |
| **10** | “Feeling on guard” | 2.68 |
| **11** | “Avoiding thoughts or feelings associated with the traumatic or hurtful events” | 2.68 |
| **12** | “Trouble sleeping” | 2.60 |
| **13** | “Difficulty concentrating” | 2.60 |
| **14** | “Feeling jumpy, easily startled” | 2.53 |
| **15** | “Feeling irritable or having outbursts of anger” | 2.53 |
| **16** | “Unable to feel emotions” | 2.50 |

Response options: 1 = “Not at all”, 2 = “A little”, 3 = “Quite a bit”, and 4 = “Extremely.”

**Depression and anxiety symptoms (1 = Not at all, 2 = A little, 3 = Quite a bit, 4 = Extremely)**

| **#** | **Depression Symptoms** | **Average Score** | **Anxiety Symptoms** | **Average Score** |
| --- | --- | --- | --- | --- |
| 1 | “Worry too much about things” | **3.49** | “Feeling tense or keyed up” | **3.13** |
| 2 | “Feeling sad” | **3.40** | “Faintness, dizziness, or weakness” | **2.73** |
| 3 | “Loss of interest in things you previously enjoyed doing” | **3.04** | “Bodily pain from distress/tension” (Investigator developed) | **2.66** |
| 4 | “Feeling hopeless about the future” | **2.72** | “Headaches” | **2.57** |
| 5 | “Feeling low in energy or slowed down” | **2.68** | “Nervousness or shakiness inside” | **2.55** |
| 6 | “Poor appetite” | **2.66** | “Feeling fearful” | **2.52** |
| 7 | “Feeling lonely” | **2.65** | “Heart pounding or racing” | **2.48** |
| 8 | “Feeling everything is an effort” | **2.62** | “Trembling” | **2.47** |
| 9 | “Feeling of worthlessness” | **2.60** | “Spell of terror or panic” | **2.42** |
| 10 | “Difficulty falling asleep or staying asleep” | **2.55** | “Feeling restless or can’t sit still” | **2.35** |
| 11 | “Crying easily” | **2.52** | “Suddenly scared for no reason” | **2.25** |
| 12 | “Feeling no interest in things” | **2.34** |  |  |
| 13 | “Feelings of being trapped or caught” | **2.40** |  |  |
| 14 | “Blaming yourself for things” | **2.10** |  |  |

Response options: 1 = “Not at all”, 2 = “A little”, 3 = “Quite a bit”, and 4 = “Extremely.”

**Investigator-developed items (1 = Not at all, 2 = A little, 3 = Quite a bit, 4 = Extremely)**

| **#** | **Item** | **Average Score** |
| --- | --- | --- |
| 1 | “Feeling humiliated/subhuman” | 2.69 |
| 2 | “Bodily pain from distress/tension” ^j^ | 2.66 |
| 3 | “Feeling disrespected” | 2.54 |
| 4 | “Feeling helpless” | 2.47 |

Response options: 1 = “Not at all”, 2 = “A little”, 3 = “Quite a bit”, and 4 = “Extremely.”
