# Supplementary material for: Systematic human rights violations, traumatic events, daily stressors and mental health of Rohingya refugees in Bangladesh
Source: Confl Health. 2020 Aug 20;14:60. doi: 10.1186/s13031-020-00306-9 (PMC7441657; doi:10.1186/s13031-020-00306-9)
Supplement: Supplementary file 5 — Additional file 5. Interpretation of Mental Health Scales. Description of data: Additional information regarding the scoring, interpretation, and limitations of the mental health measures used. [file 13031_2020_306_MOESM5_ESM.docx]

**Additional File 5: Interpretation of Mental Health Scales**

**PTSD Symptoms**

Although the PTSD subscale of the HTQ has not been validated for use with the Rohingya population, a composite cut-off score of >2.5 on the PTSD subscale of the HTQ has typically been used to indicate scores that are diagnostic of PTSD [18]. Instructions for analysis of the HTQ recommend using this cut-off score even in populations where the scale has not been validated; however, they also warn that some individuals with scores <2.5 could likely meet PTSD criteria [18]. Using the cut-off score of >2.5, **61.2% of participants endorsed posttraumatic stress symptoms typically diagnostic of PTSD**, with the average score for all participants being 2.80. However, these results should be considered with caution; because this instrument has not been normed and validated for use with this population other factors may explain such results (e.g. translation limitations, a tendency to over endorse items linked to resource expectations or other anticipated outcomes, interviewers encouraging endorsement of higher scores for similar reasons). However, such alternative explanations are unlikely considering steps the research team took in advance to address such concerns. In addition, issues such as stigma associated with mental health symptoms, common in Rohingya communities, suggests a bias towards *under* not over-endorsing of symptoms [8].

**Depression and Anxiety Symptoms**

Anxiety and depression symptom items were combined to provide a composite distress score. The higher the total score, the more likely it is that the respondent is experiencing significant emotional problems. Although the HSCL-25 has not been validated for the Rohingya population, a composite cut-off score of 1.75 for the combined anxiety and depression sub-scales has typically been used to indicate scores that are “checklist positive for some type of unspecified emotional distress” related to anxiety and depression [18].^i^ Instructions for analysis of the HSCL-25 recommend using this cut-off score, even in populations where the scale has not been validated [18]. Using this cut-off score, **84.0% of respondents endorsed anxiety and depression symptoms typically indicative of emotional distress**, with average score for all participants being 2.64. However, as mentioned above, these results should be used with caution, as this instrument has not been normed and validated for use with this population. The combined anxiety and depression subscales were used, rather than the depression subscale alone. This was done to more comprehensively capture the mental health symptoms experienced by Rohingya refugees, as represented by the high average endorsement rates of both anxiety and depression symptoms.
