# Supplementary material for: Systematic human rights violations, traumatic events, daily stressors and mental health of Rohingya refugees in Bangladesh
Source: Confl Health. 2020 Aug 20;14:60. doi: 10.1186/s13031-020-00306-9 (PMC7441657; doi:10.1186/s13031-020-00306-9)
Supplement: Supplementary file 6 — Additional file 6. Functioning Difficulty Items. Description of data: Full text of functioning items and associated average scores. [file 13031_2020_306_MOESM6_ESM.docx]

**Additional File 4: Functioning Difficulty Items**

**Functioning difficulties (1 = Not at all, 2 = A little, 3 = Quite a bit, 4 = Extremely)**

| # | **Item** | **Average Score** |
| --- | --- | --- |
| **1** | **Daily Tasks**: “How difficult is it for you to perform daily tasks? For Women: For example, tasks like cooking, caring for children, carrying water, etc. For Men: For example, tasks like working to earn money, collecting items from the market, collecting firewood, etc.” | 2.87 |
| **2** | **Hygiene:** “How difficult is it for you to care for your hygiene? For example, by bathing, washing hands, brushing teeth, washing clothes, etc.” | 2.67 |
| **3** | **Social:** “How difficult is it for you to engage in social activities? For example, activities like meeting with friends or family to spend time together.” | 2.39 |
| **4** | **Religious:** “How difficult is it for you to engage in religious activities? For Women: For example, activities like praying Namaz, reciting the Quran, etc. For Men: For example, activities like praying Namaz, going to musjid, reciting the Quran, etc.” | 1.60 |

Response options: 1 = “Not at all”, 2 = “A little”, 3 = “Quite a bit”, and 4 = “Extremely.”
