# Supplementary material for: Systematic human rights violations, traumatic events, daily stressors and mental health of Rohingya refugees in Bangladesh
Source: Confl Health. 2020 Aug 20;14:60. doi: 10.1186/s13031-020-00306-9 (PMC7441657; doi:10.1186/s13031-020-00306-9)
Supplement: Supplementary file 7 — Additional file 7. Full Data from Final Regression Models. Description of data: Full data for the final regression models presented in the manuscript. [file 13031_2020_306_MOESM7_ESM.docx]

**Additional File 7: Full Data from Final Regression Models**

**Model 1. predicting PTSD symptoms R^2^ = .583, F (8, 469) = 82.05 p < .001**

| **Variables** | **B** | **Std. Error** | **β** | **t** | **Sig.** | **95% CI** |
| --- | --- | --- | --- | --- | --- | --- |
| **1. Bangladesh daily stressors** | .000087 | .012 | .000 | .007 | .994 | -.023, .024 |
| **2. Myanmar daily stressors** | .036 | .014 | .000 | 2.461 | .014* | .007, .064 |
| **3. Sex** | -.151 | .061 | -.094 | -2.488 | .013* | -.270, -.032 |
| **4. Systematic human rights violations** | .041 | .015 | .095 | 2.756 | .006** | .012, .070 |
| **5. Age** | .006 | .002 | .097 | 3.147 | .002** | .002, .010 |
| **6. Trauma history** | .038 | .007 | .185 | 5.417 | .000** | .024, .051 |
| **7. Feeling humiliated/subhuman** | .195 | .028 | .313 | 6.885 | .000** | .139, .251 |
| **8. Feeling helpless** | .246 | .028 | .366 | 8.807 | .000** | .191, .301 |

*p < .05, **p < .01

**Model 2. Predicting emotional distress R^2^ = .382, F (6, 471) = 48.47 p < .001**

| **Variables** | **B** | **Std. Error** | **β** | **t** | **Sig.** | **95% CI** |
| --- | --- | --- | --- | --- | --- | --- |
| **1. Sex** | .060 | .069 | .037 | .868 | .386 | -.076, .195 |
| **2. Bangladesh daily stressors** | .039 | .014 | .105 | 2.725 | .007** | .011, .068 |
| **3. Age** | .007 | .002 | .109 | 2.911 | .004** | .002,.011 |
| **4. Systematic human rights violations** | .069 | .018 | .160 | 3.890 | .000** | .034, .104 |
| **5. Myanmar daily stressors** | .145 | .016 | .337 | 9.029 | .000** | .113, .176 |
| **6. Trauma history** | .070 | .008 | .341 | 8.461 | .000** | .053, .086 |

*p < .05, **p < .01

**Model 3. Predicting functioning difficulties R^2^ = .451, F (6, 483) = 66.26 p < .001**

| **Variables** | **B** | **Std. Error** | **β** | **t** | **Sig.** | **95% CI** |
| --- | --- | --- | --- | --- | --- | --- |
| **1. Age** | -.004 | .008 | -.017 | -.471 | .638 | -.020, .012 |
| **2. Trauma history** | .025 | .030 | .033 | .834 | .405 | -.034, .085 |
| **3. Sex** | -.403 | .218 | -.065 | -1.850 | .065 | -.831, .025 |
| **4. PTSD symptoms** | .539 | .270 | .140 | 1.996 | .047* | .008, 1.069 |
| **5. Depression symptoms** | 1.476 | .299 | .362 | 4.939 | .000** | .889, 2.063 |
| **6. Bangladesh daily stressors** | .482 | .052 | .336 | 9.258 | .000** | .379, .584 |

*p < .05, **p < .01
